# 

**Published:** 1882-05

**Authors:** 


### CHICAGO MEDICAL Journal and Examiner

EDITORS:

N. S. DAVIS, M.D., LL.D. JAS. NEVINS HYDE, A.M., M.D.

DANIEL R. BROWER, A.M., M.D.

Chicago Medical Press Association. Aug. 2d 1875.

CHICAGO.

*PUBLISHED BY THE CHICAGO MEDICAL PRESS ASSOCIATION,*

188 Clark Street.

S.G.O.L. INDEXED

☞ **Read the Notice of this Journal among Advertisements.**

